# Tackling adverse health effects of climate change and migration through intersectoral capacity building in Sub-Saharan Africa

**DOI:** 10.3399/bjgpopen20X101065

**Published:** 2020-04-16

**Authors:** Charlotte Scheerens, Ilse Ruyssen, Sunanda Ray, An De Sutter, Wouter Vanhove, Els Bekaert, Bob Mash, Peter Decat, Jan De Maeseneer

**Affiliations:** 1 Department of Public Health and Primary Care, Ghent University, Ghent, Belgium; 2 Department of Geriatrics, University of California San Francisco, San Francisco, US; 3 Department of Economics, Ghent University, Belgium, UNU-CRIS Belgium; 4 Department of Community Medicine (retired), University of Zimbabwe College of Health Sciences, Harare, Zimbabwe; 5 Laboratory of Tropical and Subtropical Agriculture and Ethnobotany, Ghent University, Ghent, Belgium; 6 Division of Family Medicine and Primary Care, Faculty of Medicine and Health Sciences, Stellenbosch University, Stellenbosch, South Africa

**Keywords:** Climate change, capacity building, family physicians, primary healthcare, general practice

The international community increasingly acknowledges that climate change is not only spurring environmental degradation and health deterioration,^1^ it is also an important direct and indirect driver of migration, conflict, and human rights violations. This is particularly true for Sub-Saharan Africa (SSA), the region where people — in contrast to high-income countries and high energy consuming industries, which emit the most greenhouse gasses on a global scale — are relatively more vulnerable to climate change, because their precarious socioeconomic status, lack of resources, and weak health systems constrain their adaptation capacity.^2^ Still, debate and research on the explicit interactions between climate change, migration, and health are scarce, and prevailing research and policies disproportionally neglect the voice of the global South, such as that of SSA.

During the African conference of the World Organisation of Family Doctors (WONCA) and the African Primary Care and Family Medicine Education Network (Primafamed) meeting in Kampala, Uganda (4–6, June 2019), we organised focus groups with primary health care experts and family physicians (FPs) from twelve SSA countries, to explore these interactions. Although the climate research community underrepresents FPs, the latter are excellent sources of information. Their position in the centre of the community gives them insight into the health of the population and the underlying social and environmental determinants of health. An international interdisciplinary research team will analyse the data from the focus groups, and document the complex and accumulating impacts of climate change and migration on individuals’, families’, and communities’ livelihoods and health. The following quote is one of the many illustrating the complexity of these interactions: a participant explained how 2019’s Cyclone Idai, which had catastrophic outcomes in Malawi, Mozambique, and Zimbabwe, set in motion a cycle of events affecting people’s lives and further weakened the region’s vulnerable health system:

*‘We’ve had cyclones before, but this one was really horrific. What pains me … is the government did nothing about warning … people to move to higher safer grounds. The cyclone struck down the roads and people’s houses were swept* [away] *overnight, some communities vanished completely and … bodies could not be retrieved...* [there was] *a lot of anxiety, depression amongst relatives in* [countries] *with very weak health system[s] right now*.

*To … help those people was not easy. We* [have] *a high prevalence of HIV ... some whose homesteads were swept away … did not have access to their antiretroviral medication. The government took too long to respond … people* [did not have access to] *clean water, which means a whole lot of water-borne diseases coming from that place. My main worry is it is going to take a long time before the infrastructure is repaired and the clinics are revived. What if another catastrophe hits the same area? You can imagine people migrating from that region to* [another] *one. How is that health system going to cope? There* [will] *be infectious diseases arising from that. And we have lost a lot of doctors, also they leave the country to look for greener pastures*.‘

In the aftermath of Cyclone Idai, Mozambique had more than 130 000 homeless people, 112 000 damaged or destroyed homes, 1.77 million acres of damaged or destroyed crops, and 1420 notified cases of cholera. At least 598 died (including two confirmed from cholera) in Mozambique and 181 in Zimbabwe. In one Zimbabwean town, Chipinge, the district’s water supply was severely damaged, leaving more than 30 000 people without access to safe drinking water. In Malawi, 868 900 people were affected by the cyclone, including 87 000 internally displaced persons, of whom more than 60% were women and children.^3^

Focus group participants stressed the need for more intersectoral action between primary health care teams, agriculture, and education to address the adverse outcomes of climate change on migration and health. For example, intersectoral Emergency Response Teams should better integrate FPs and government medical officers, since they interact closely with communities of greatest need. Furthermore, fairer political and trade partnerships with the global North, alongside ethical agricultural and food production policies form a precondition for SSA to be able to cope with the implications of future climate crises. High income countries should, meanwhile, take up their unquestionable responsibility to reduce greenhouse gas emissions urgently and drastically.

Since the international community is increasingly shifting attention to addressing the health impact of climate change, investment in interprofessional teams is also extremely relevant. Recently, large donors seem to be inclined to finance Community Health Worker (CHW)-focused capacity building programs. A recent editorial by the Director-General of the WHO even states that ’*CHWs serve as the first point of contact for people needing healthcare; they are the functional link between communities and health facilities, such as hospitals’*.^4^ While investing in CHWs is indeed a relatively cheap way of delivering some aspects of primary care coverage, providing comprehensive quality primary care requires an interprofessional primary healthcare team. In fact, evidence shows that scaling up capacity-building of interprofessional teams (involving FPs, nurses, social workers, CHWs, etc) working in clinics and health centres in the community, is indispensable to make the most effective difference in the context of universal health coverage and wellbeing for all (Sustainable Development Goal 3).^5,6^

Finally, the Expert Panel on Effective Ways of Investing in Health, advising the European Commission, calls for a more integrated approach in the face of climate change: *’Migration (and refugee-crisis), climate change and capacity building (for healthcare, education, food production …) in the global South, are strongly interrelated. EU policy could address these issues in an integrated and more comprehensive way, looking at push- and pull-factors, socio-economic and ecological drivers.’* ^7^ The first results of our qualitative research underpin the need for broad political commitment through global inter-sectoral action in order to tackle the complex and intertwined cause–effect relationship of health, migration, and climate change.
